# The Predictive Role of ctDNA and CTCs in Patients with Advanced Non-Small Cell Lung Cancer Receiving Immunotherapy: A Systematic Review and Meta-Analysis

**DOI:** 10.3390/ijms27125518

**Published:** 2026-06-18

**Authors:** Andrea C. Kakouri, Maria Spiliotaki, Constantinos Deltas, Gregory Papagregoriou, Haris Charalambous

**Affiliations:** 1 biobank.cy Center of Excellence in Biobanking and Biomedical Research, University of Cyprus, Nicosia 2029, Cyprus; kakouri.andrea@ucy.ac.cy (A.C.K.); spiliotaki.maria@ucy.ac.cy (M.S.); deltas@ucy.ac.cy (C.D.); 2 University of Nicosia Medical School, Nicosia 2408, Cyprus; 3 Medical Oncology Department, Bank of Cyprus Oncology Centre, Acropoleos Ave 32, Nicosia 2006, Cyprus

**Keywords:** NSCLC, liquid biopsy, biomarkers, immunotherapy, response, ctDNA, CTCs

## Abstract

Circulating tumour DNA (ctDNA) and circulating tumour cells (CTCs) have emerged as promising non-invasive biomarkers for the immunotherapy response in advanced non-small cell lung cancer (NSCLC). However, their clinical utility remains uncertain due to variability in findings across studies. We conducted a systematic review and meta-analysis of studies from 2014 to 2024, assessing the predictive value of ctDNA and CTCs in advanced NSCLC patients receiving immunotherapy. Primary outcomes were progression-free survival (PFS) and overall survival (OS). Forty-four studies were included (28 ctDNA, 16 CTC cohorts). High baseline ctDNA was associated with worse OS (pooled HR = 1.38, 95% CIs: 1.17–1.63), while baseline CTC detection predicted worse PFS (pooled HR of 3.65 (95% CIs: 1.58–8.41) and OS (pooled HR = 2.30, 95% CIs: 1.54–3.44). An on-treatment ctDNA decrease or clearance was associated with improved PFS (pooled HR = 0.34, 95% CIs: 0.25–0.47) and OS (pooled HR = 0.33, 95% CIs: 0.24–0.44). Evidence for other ctDNA- and CTC-derived biomarkers (blood tumour mutational burden, genomic alterations, dynamic CTC changes, and CTC PD-L1 expression) was limited or inconsistent. The interpretation of these findings is limited by heterogeneity in assay platforms, biomarker definitions, the analytical threshold, and sampling timepoints across studies. While ctDNA and CTCs show significant potential as predictive biomarkers in advanced NSCLC, further validation is needed in larger prospective studies using standardized assays. At present, ctDNA and CTC monitoring can complement but cannot replace radiological assessments in guiding immunotherapy decisions for NSCLC patients.

## 1. Introduction

### 1.1. The Burden of Lung Cancer and the Role of Immunotherapy

Lung cancer (LC) remains the leading cause of cancer-related death worldwide (World Health Organization, 2020 Statistics), with non-small cell lung cancer (NSCLC) accounting for approximately 85% of all LC cases. More than half of NSCLC patients are diagnosed with metastatic disease and have a very poor prognosis, with only about 3% surviving five years [1]. The treatment of advanced NSCLC traditionally relied on a combination of chemotherapy and radiotherapy, with targeted therapies reserved for the small subset of patients with actionable driver mutations. Over the past decade, immunotherapy has revolutionized the therapeutic landscape, offering new hope for patients with metastatic disease and therefore poor prognosis. The introduction of immune checkpoint inhibitors (ICIs), targeting immune regulatory pathways such as programmed cell death protein 1 (PD-1) and its ligand PD-L1, has significantly improved survival outcomes in advanced NSCLC. ICIs have become the standard of care, either as monotherapy or in combination with chemotherapy, depending on PD-L1 expression levels and the disease stage. For patients with high PD-L1 expression (≥50%), ICIs as monotherapy have demonstrated long-term survival benefits, with five-year survival rates reaching up to 30% [2].

### 1.2. The Unmet Need for Predictive Biomarkers

However, for patients without high PD-L1 expression, the use of ICIs with or without chemotherapy has provided less benefit, with 5-year survival rates being less than 15% [3], hence the need for predictive biomarkers to guide therapy selection and maximize benefits. Other challenges for the use of ICIs include the high cost of ICIs, toxicity, which in some patients is severe, requiring immunosuppression with high-dose steroids and sometimes other immunosuppressants, and the difficulty to predict and monitor treatment responses. Monitoring treatment efficacy is currently based on clinical and radiological assessments. The interpretation of imaging in NSCLC patients treated with ICIs can sometimes be challenging due to pseudoprogression, a phenomenon characterized by an initial increase in tumour size, followed by tumour regression. Furthermore, patients generally have to wait at least 6–9 weeks for repeat CT scans to be undertaken to know if treatment is effective or not.

Predictive biomarkers are important with all oncology treatments in order to avoid use in patients not likely to benefit or to identify patients not benefiting during treatment, allowing earlier treatment changes and reducing both clinical and financial toxicity. Given these limitations, there is considerable interest in minimally invasive biomarkers that can provide real-time insights into the tumour burden and treatment response during immunotherapy. Liquid biopsy approaches, particularly ctDNA and CTCs, have emerged as promising tools for this purpose.

### 1.3. ctDNA

ctDNA is released into the bloodstream from primary and/or metastatic tumours through processes such as apoptosis, necrosis, autophagy, and necroptosis. It represents the tumour-derived fraction of cell-free DNA (cfDNA), typically accounting for approximately 10% of the total cfDNA, depending on the tumour type and burden [4]. They are short DNA fragments, typically ~180–200 base pairs in length, that can be isolated from plasma or serum and analysed for the identification and quantification of tumour-specific alterations in oncogenes and tumour-suppressor genes [5]. ctDNA analysis allows for the rapid and accurate detection of de novo and resistant-to-treatment genetic alterations, as well as the real-time monitoring of treatment responses. In contrast to solid tumour biopsies, LB is minimally invasive and captures more of the heterogeneity of tumours, allowing the detection of genetic alterations responsible for treatment resistance, which could be missed by tissue genotyping [3].

In recent years, LB based on ctDNA analysis has shed a new light on the molecular diagnosis, prognosis, and monitoring of cancer, including NSCLC [6,7]. Two main analytical approaches are used for its detection: (a) tumour-informed and (b) tumour-agnostic assays. Tumour-informed assays utilize prior sequencing of the primary tumour to create patient-specific panels targeting known genomic alterations, offering superior accuracy and lower limits of detection. These personalized panels have achieved remarkable sensitivity improvements, with limits of detection as low as 0.00037%, depending on the number of variants analysed [5]. However, tumour-informed approaches are limited by 3- to 4-week turnaround times and tissue requirements, creating challenges for early-stage NSCLC patients with only small biopsies, those with complete responses lacking residual tissue, and metastatic patients with inaccessible or insufficient samples. Tumour-agnostic approaches provide faster turnaround times of 7–10 days and do not require prior tumour sequencing, making them more practical for routine monitoring. While these assays may have higher limits of detection and can produce false-positive results from clonal haematopoiesis, they can detect previously unknown genomic alterations that arise during the course of disease or alterations missed by tissue biopsy due to sampling limitations or spatial tumour heterogeneity. This capability allows tumour-agnostic assays to capture spatial and temporal tumour heterogeneity, identifying new mutations that occur across different tumour sites or emerge as resistance mechanisms during treatment. Next-generation tumour-agnostic platforms incorporating machine learning and multi-omics approaches, including methylation and fragmentomic profiling, are demonstrating improved sensitivity for ctDNA detection while maintaining this broader mutational coverage [5]. Methylation profiling identifies cancer by detecting unique DNA methylation patterns, which differentiate proliferating from healthy-state cells. Fragmentomic profiling examines the size and distribution of DNA fragments in blood, as tumour-derived DNA fragments are typically shorter and differently patterned than healthy cfDNA. Together, these methods provide additional non-genomic signals to enhance the sensitivity of ctDNA detection.

### 1.4. CTCs

CTCs are cancer cells shed from a solid tumour to enter the bloodstream, where they can potentially establish metastatic lesions at distant sites [8]. CTCs exhibit a range of physical and biological properties influenced by their tissue of origin and metastatic potential. Their diameters vary significantly, from 10 μm in small cell lung cancer to over 100 μm in prostate cancer. CTCs retain protein profiles specific to the tumour type, such as epithelial adhesion markers and organ-specific proteins. CTCs are classically defined as nucleated cells expressing cytokeratin and lacking the blood cell marker CD45. However, this definition does not capture all tumour-derived circulating cells. CTCs display substantial morphological heterogeneity in shape and size, with some CTCs showing low or absent cytokeratin expression, indicating a potential transition to a more invasive mesenchymal phenotype [9]. This heterogeneity complicates CTC detection but provides valuable insights into tumour progression and the treatment response. Although CTCs have been known for over 150 years, recent technological advancements, such as the anti-EpCAM antibody-based CELLSEARCH platform, have enabled their clinical application. However, challenges, such as a reduction in EpCAM expression due to epithelial–mesenchymal transition (EMT), make it difficult to capture all CTC subtypes. New techniques, including size- and density-based isolation systems, have proven effective in capturing CTCs independent of epithelial markers. CTC quantification has shown potential as an indicator of tumour burden and aggressiveness, and emerging research is exploring their role in cancer detection. Studies are ongoing to better understand the diverse subpopulations of CTCs and their implications for cancer diagnosis and prognosis [10].

### 1.5. Current Clinical Role of ctDNA and CTC Biomarkers

Previous reviews and guidelines have examined the role of ctDNA and CTCs in NSCLC management, including diagnosis, prognosis, and treatment monitoring. For diagnosis, ctDNA shows high specificity for tumour-associated mutations but limited sensitivity in early-stage disease, improving in metastatic settings where concordance with tissue NGS is high [4,11]. Accordingly, supplementary tissue biopsies are used when feasible for comprehensive molecular profiling [12,13,14]. Regarding prognosis and treatment monitoring, the clinical value of ctDNA has been more distinctly emphasized. It was previously shown that ctDNA-based minimal residual disease (MRD) detection can predict relapse and survival in early-stage NSCLC patients when measured during or after definitive therapy, as well as at post-operative and post-chemotherapy timepoints [15,16]. Wang et al. also reported that an early ctDNA reduction was associated with improved OS, PFS, and ORR, while baseline ctDNA detection was not significantly associated with outcomes [17]. Further supporting ctDNA’s predictive significance, ctDNA-derived *EGFR* mutations have been linked to improved overall survival (OS) in advanced NSCLC patients treated with EGFR tyrosine kinase inhibitors (TKIs) [18]. Complementing these findings, significant reductions in or the complete clearance of ctDNA levels following ICI treatment have been associated with improved progression-free survival (PFS) and OS [19,20].

Similarly, there is evidence to suggest that CTCs remain a valuable biomarker for NSCLC. Several studies suggest that CTCs can differentiate lung cancer patients from controls [21] and, alongside ctDNA, can accurately identify early-stage NSCLC [15]. The detection of CTCs before and after surgery in early NSCLC is linked to an increased risk of relapse and death, reinforcing their role in monitoring MRD at different stages [15,22]. In advanced NSCLC, high CTC counts before chemotherapy or TKIs are associated with shorter PFS [23]. Studies also show that the detection of CTCs correlates with poorer OS, particularly in early-stage NSCLC [24]. Additionally, CTC counts greater than two per 7.5 mL of blood were found to predict poor outcomes in advanced NSCLC [25]. Meta-analyses show that CTC detection is histology-independent but correlates with lymph node metastasis and poorer OS [26]. A separate review on immunotherapy-treated advanced NSCLC found that a high blood tumour mutational burden (bTMB) and low PD-L1 levels on CTCs predict better responses, with PD-L1-expressing CTCs associated with worse outcomes [20].

### 1.6. Aim of the Study

Despite growing evidence supporting the clinical utility of ctDNA and CTCs in NSCLC, their predictive value in patients receiving ICIs remains incompletely defined. Previous reviews have primarily focused on diagnostic applications, molecular profiling, minimal residual disease detection, or broader NSCLC populations without quantitatively synthesizing the available evidence in immunotherapy-treated advanced disease. Therefore, the objective of this systematic review and meta-analysis was to evaluate the predictive significance of baseline levels and early on-treatment changes in ctDNA and CTCs in adults with advanced or metastatic NSCLC receiving ICI-based therapy. The primary endpoints were PFS and OS.

## 2. Methods

This systematic review and meta-analysis was conducted and reported in accordance with the Preferred Reporting Items for Systematic Reviews and Meta-Analyses (PRISMA) 2020 guidelines. A completed PRISMA 2020 checklist [27] is provided in the Supplementary Materials.

### 2.1. Protocol and Registration

No protocol was prospectively registered in PROSPERO or another publicly accessible registry prior to conducting this review.

### 2.2. Eligibility Criteria

To be eligible for inclusion, studies had to satisfy the following criteria: (i) be observational studies (prospective or retrospective) and randomized controlled trials, (ii) refer to humans, (iii) include patients with advanced and/or metastatic NSCLC treated with ICIs of any type, either monotherapy or combinatorial therapy, (iv) be studies with the documented collection of ctDNA, CTCs, and outcome data (serial or non-serial), such as progression-free survival (PFS), and overall survival (OS). All methods of ctDNA and CTC detection and analysis were allowed, given the lack of a gold standard and of direct comparisons between the various methods. Studies were eligible only if they were written in English. Exclusion criteria included the following: (i) review articles, editorials, comments, and letters to the editor, (ii) ongoing studies with results not presented or published at the time of the literature search. The authors and institutions were reviewed to avoid repetition of databases (duplicated studies). 

### 2.3. Information Sources

PubMed (https://pubmed.ncbi.nlm.nih.gov) and Web of Science (https://webofscience.com) were searched from 1 January 2014 through 31 August 2024.

### 2.4. Search Strategy

For each database, the following field tags, strings, and Boolean operators were used: “PD-1” OR “PD-L1” OR “CTLA-4” OR “ipilimumab” OR “tremelimumab” OR “nivolumab” OR “pembrolizumab” OR “lambrolizumab” OR “atezolizumab” OR “avelumab” OR “durvalumab” OR “immune checkpoint inhibitor” OR “immune checkpoint inhibitors” OR “ICI” OR “ICIs” OR “immune checkpoint blocker” OR “immune checkpoint blockers” OR “ICB” OR “ICBs”) AND (“ctDNA” OR “circulating tumour DNA” OR “circulating tumour DNA” OR “CTC” OR “CTCs” OR “circulating tumour cell” OR “circulating tumour cells”) AND (“lung cancer” OR “non-small cell lung cancer” OR “lung adenocarcinoma” OR “NSCLC”). Spelling issues were addressed by using established abbreviations for every search term.

### 2.5. Selection Process

Two investigators (ACK, MS) independently reviewed titles and abstracts, followed by full-text assessments of potentially eligible studies. Disagreements were resolved through discussion. No automation tools were used. During the entire selection process, none of the authors was blinded to the source of the publications, the authors, or any other details.

### 2.6. Data Types and Collection Process

Data extraction was performed by two reviewers (ACK, MS), with disagreements resolved through consensus or, when required, through consultation with a third reviewer (HC). The following data were retrieved from the studies included: author, publication year, country in which the study was conducted, study type (prospective/retrospective), number of patients included in the analysis, percentage of male and female patients, age distribution, NSCLC subtype, stage, smoking status, type of immunotherapy, method used for ctDNA/CTC detection and/or measurement, CTC markers evaluated, and outcome (PFS, OS). More specifically, ctDNA and CTC metrics and on-treatment change definitions, including the maximal allele fraction, tumour fraction, concentration, variant clearance, copy-number burden, CTC counts, and PD-L1 on CTCs, were collected at predefined timepoints (baseline and post-treatment) together with analysis metrics and effect measures if reported, such as hazard ratios (HRs) with 95% confidence intervals (CIs) for PFS and OS and odds or risk ratios for objective responses. We prespecified PFS as the primary outcome and OS as secondary.

### 2.7. Quality Assessment

Risk of bias was assessed with QUIPS, a tool designed for prognostic factor studies, by two independent reviewers, with disagreements resolved through consensus. Studies were judged on participation, attrition, prognostic factor measurement, outcome measurement, confounding, and analysis and reporting, and overall risk was determined based on the worst important domain. “Low” was assigned when methods were clearly described and appropriate, with concerns unlikely to alter the biomarker–outcome association. “Moderate” was assigned when some limitations were present but meaningful distortion of the effect was unlikely. “High” was assigned when serious shortcomings could plausibly distort the association. The Level of Evidence (LoE) for each study was assigned according to the Oxford Centre of Evidence-Based Medicine (OCEBM) 2011 framework (Levels I–IV), with higher levels indicating stronger study designs for prognostic inference. For ctDNA and CTCs, a clear assay description, timing at baseline and within two cycles, prespecified thresholds or definitions for the molecular response, and standard outcome definitions for PFS, OS, and RECIST responses were required for low-risk ratings. A low risk for confounding was assigned only when models were adjusted for performance status, PD-L1, line of therapy, histology, smoking, and metastatic burden. Sensitivity summaries were planned so that studies at a high risk in confounding or analysis would be downweighed or excluded. Reporting completeness was assessed using the REMARK checklist [28], and levels of clinical evidence were annotated according to ESMO criteria [29]. Any disagreements were resolved through consensus or, if needed, by a third reviewer (HC).

### 2.8. Measurement of Treatment Effect

For time-to-event outcomes, the preferred effect measure was a HR with 95% CIs. Study-reported HRs for progression-free survival (PFS) and overall survival (OS) were extracted where available; otherwise, the direction of the effect was recorded. PFS and OS were accepted as defined by each study, typically from treatment initiation to documented progression and from treatment initiation to death, respectively. Where effect estimates were sufficiently comparable, pooled analyses were performed as prespecified; otherwise, findings were synthesized narratively.

### 2.9. Statistical Analysis

Meta-analysis was performed for studies reporting HRs with 95% CIs for PFS and OS. Studies that did not report HRs with 95% CIs or that presented outcomes only as *p*-values, median survival differences, response rates, or non-comparable effect measures were not included in the quantitative pooling and were synthesized narratively. Separate pooled analyses were conducted for ctDNA and CTC studies according to the biomarker construct and timing of assessment, and pooling was restricted to studies evaluating comparable constructs. When multiple estimates were available from the same study within a given endpoint and biomarker group, multivariable estimates were preferentially retained over univariable estimates, and only one estimate per cohort was included in each pooled analysis to avoid non-independence. Random-effects models were fitted using restricted maximum likelihood (REML), which accounts for both within-study variance and between-study heterogeneity. A random-effects approach was selected because clinical and methodological heterogeneity was expected across studies, including differences in assay platforms, biomarker thresholds, treatment regimens, and sampling timepoints. Between-study heterogeneity was assessed using Cochran’s Q test and the I^2^ statistic. Cochran’s Q evaluates whether observed variability in effect estimates exceeds that expected by chance alone, whereas I^2^ quantifies the proportion of total variability attributable to between-study heterogeneity rather than sampling error. An assessment of publication bias and small-study effects was planned only for pooled groups including at least 10 studies, using funnel plots and trim-and-fill analysis. Formal testing for funnel-plot asymmetry was not performed for smaller pooled analyses because of the limited number of studies included. Statistical analysis and plot generation were performed in R studio (version 2026.04.0+562).

### 2.10. Certainty Assessment

Certainty of evidence was appraised using GRADE adapted for prognostic and predictive factor research at the outcome level. Certainty was downgraded for study limitations based on QUIPS judgments, inconsistency across studies, indirectness related to assay platforms, thresholds, and sampling timepoints, imprecision arising from small cohorts and wide confidence intervals, and publication bias when assessable. Final certainty ratings reflected the totality of evidence for each contrast and outcome.

### 2.11. Ethics Statement

Not applicable. This review used published data.

## 3. Results

### 3.1. Study Selection

**Figure 1:**
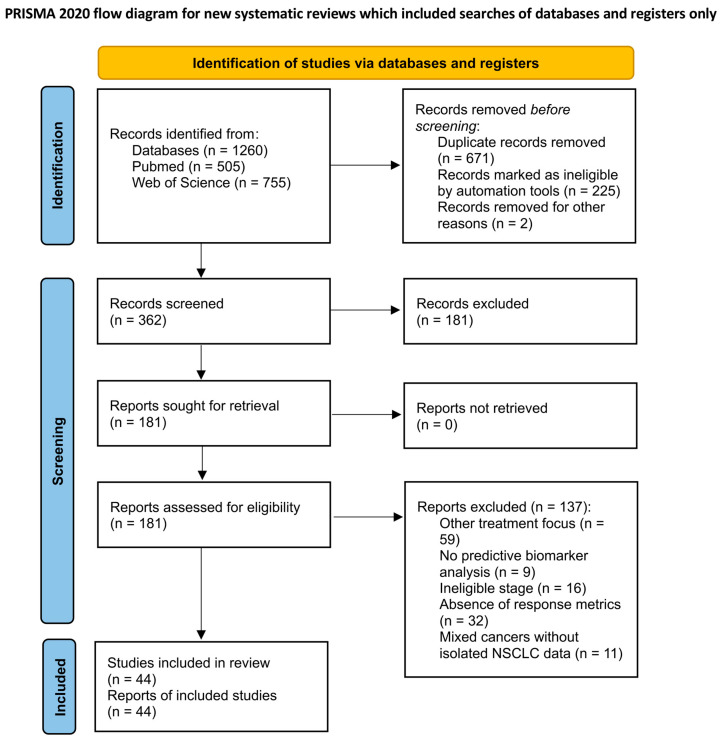
The study selection process is presented in the PRISMA flow diagram.

Database searches yielded 362 records after deduplication, 232 from PubMed and 130 from Web of Science (Figure 1), of which 181 were excluded after title and abstract screening. Of the remaining 181 full-text reports, 137 were excluded for reasons including irrelevant treatment focus, no predictive biomarker analysis, ineligible stage, absence of standard response metrics, or mixed cancers without extractable NSCLC data. Forty-four studies were included, comprising 28 ctDNA cohorts (*n* = 2259) and 16 CTC cohorts (*n* = 743). Detailed reasons for full-text exclusion are shown in Figure 1.

### 3.2. Study Characteristics

Included cohorts are comprised of patients with advanced or metastatic NSCLC treated with ICIs, either as monotherapy or in combination regimens. Overall, 28 ctDNA cohorts including 2259 patients and 16 CTC cohorts including 743 patients were assessed. Included ctDNA cohorts spanned tumour-informed and tumour-agnostic assays, whereas CTC studies used diverse enrichment and staining strategies. Sampling commonly occurred at baseline and again within two to nine weeks after immunotherapy initiation. Several ctDNA studies evaluated the molecular response using relative change thresholds such as a 50% decrease or clearance to undetectable levels, and some assessed the blood TMB or specific variants. PFS and OS were the main outcomes, although several studies also reported the RECIST response, durable clinical benefit, or objective response. Overall, included studies varied substantially in assay design, with ctDNA studies using tumour-informed and tumour-agnostic approaches and CTC studies using different enrichment, isolation, and immunostaining platforms. Characteristics and outcome definitions are summarized in Table 1 and Table 2.

### 3.3. Risk of Bias in Studies

Risk of bias was judged with QUIPS (Supplementary Table S1). Across 44 cohorts included, overall risk was “Moderate” in 31, “Low” in 10, and “High” in 3 studies. Confounding was the principal high-risk domain, with 18 studies rated “High” and 20 rated “Moderate”, reflecting a limited adjustment for key clinical variables such as the performance status, PD-L1 expression, line of therapy, histology, smoking status, and metastatic burden. Statistical analysis and reporting also contributed to risk-of-bias concerns, with 28 studies rated “Moderate” and 3 rated “High”, mainly due to small cohorts, incomplete effect reporting or non-standardized analytical approaches. The prognostic factor measurement was frequently rated “Moderate”, reflecting heterogeneity in ctDNA and CTC assay platforms, biomarker thresholds, and sampling schedules. The outcome measurement was generally low risk, whereas confounding and statistical analysis domains contributed most to concerns, with confounding rated “High” in 18 studies and “Moderate” in 20. ctDNA cohorts were predominantly “Moderate”, while CTC cohorts showed more “High”-risk judgments due to platform heterogeneity and smaller sample sizes. These patterns support the “Low” certainty rating for an early ctDNA decline and CTC-based contrasts in the GRADE assessment. Reporting quality was described with the REMARK checklist and evidence levels with ESMO criteria. Detailed study-level QUIPS assessments are provided in Supplementary Table S1.

### 3.4. Quantitative Synthesis

The meta-analysis was restricted to biomarker constructs with sufficient clinical and methodological comparability for quantitative pooling; ctDNA and CTC measures that did not meet these criteria were not pooled and were instead retained for narrative synthesis. Only studies reporting HRs with 95% CIs were eligible for quantitative synthesis. Quantitative pooling was further limited to studies assessing comparable biomarker constructs and construct definitions. Accordingly, of the 28 ctDNA studies, 16 were included in the quantitative synthesis, while of the 16 CTC studies, 6 were quantitatively pooled.

**Figure 2:**
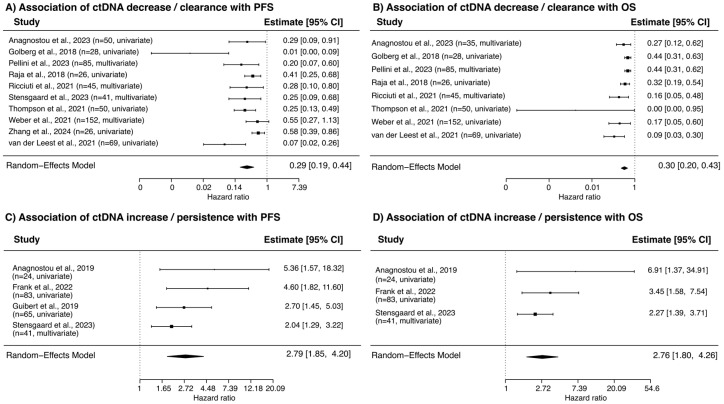
Forest plots of pooled hazard ratios (HRs) with 95% confidence intervals (CIs) for associations between on-treatment ctDNA dynamics and clinical outcomes. (**A**) ctDNA decrease/clearance and PFS [30,34,43,44,46,47,48,49,51,52]; (**B**) ctDNA decrease/clearance and OS [30,34,43,44,46,48,49,51]; (**C**) ctDNA increase/persistence and PFS [31,33,47,55]; (**D**) ctDNA increase/persistence and OS [31,33,47].

**Figure 3:**
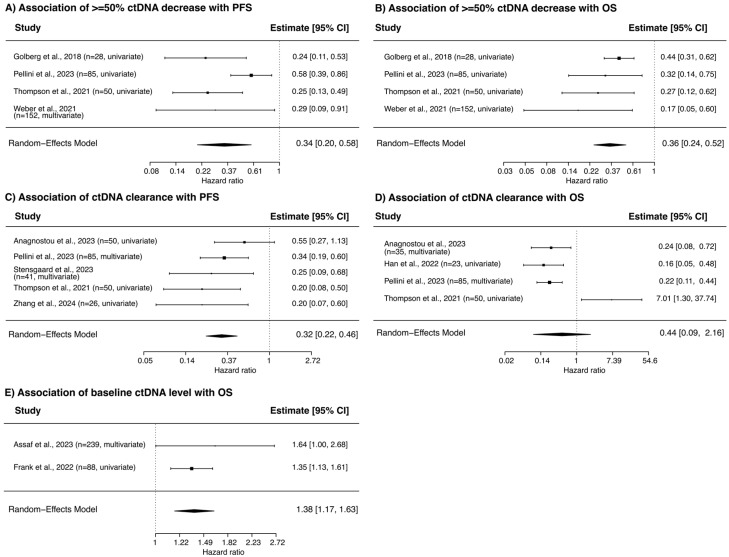
Forest plots and pooled hazard ratios (HRs) with 95% confidence intervals (CIs) for exploratory ctDNA analyses; (**A**) ≥50% ctDNA decrease and PFS [34,43,48,51]; (**B**) ≥50% ctDNA decrease and OS [34,43,48,51]; (**C**) ctDNA clearance and PFS [30,43,47,48,52]; (**D**) ctDNA clearance and OS [30,35,43,48]; (**E**) baseline ctDNA levels and OS [32,33].

For ctDNA, the most consistent pooled signal was observed for early on-treatment ctDNA decrease/clearance. For PFS, ctDNA decrease/clearance was associated with significantly improved outcomes across 10 cohorts (patient *n* = 572, pooled HR = 0.34, 95% CIs: 0.25–0.47, *p* = 3.28 × 10^−11^, I^2^ = 38.7; Figure 2A). For OS, the same construct was associated with significantly improved survival across 8 cohorts (patient *n* = 490, pooled HR = 0.33, 95% CIs: 0.24–0.44, *p* = 8.11 × 10^−14^, I^2^ = 33.8; Figure 2B). In contrast, on-treatment ctDNA increase/persistence was associated with worse outcomes, with pooled HRs of 2.79 (95% CIs: 1.85–4.20) for PFS across 4 cohorts (patient *n* = 213, *p* = 9.07 × 10^−7^, I^2^ = 25.2; Figure 2C) and 2.76 (95% CIs: 1.80–4.26) for OS across 3 cohorts (patient *n* = 148, *p* = 3.91 × 10^−6^, I^2^ = 6.5; Figure 2D). Exploratory subgroup analyses showed that the favourable association remained directionally consistent when the response was defined more specifically. A ≥50% ctDNA decrease was associated with improved OS (patient *n* = 315, pooled HR = 0.36, 95% CIs: 0.24–0.52, *p* = 5.48 × 10^−8^, I^2^ = 17.8; 4 cohorts; Figure 3B) and PFS (patient *n* = 315, pooled HR = 0.34, 95% CIs: 0.20–0.58; 4 cohorts, *p* = 6.16 × 10^−5^, I^2^ = 55.7; Figure 3A). Similarly, ctDNA clearance was associated with improved PFS (patient *n* = 252, pooled HR = 0.32, 95% CIs: 0.22–0.46 *p* = 1.57 × 10^−9^, I^2^ = 6.8; 5 cohorts; Figure 3C). The OS clearance subgroup was less precise (patient *n* = 193, pooled HR = 0.44, 95% CIs: 0.09–2.16, *p* = 0.31, I^2^ = 88.4; 4 cohorts; Figure 3D), likely reflecting smaller numbers and between-study heterogeneity. In addition, an exploratory pooled analysis of the baseline ctDNA level for OS, based on 2 cohorts (patient *n* = 327), showed that higher baseline ctDNA was associated with worse survival (pooled HR = 1.38, 95% CIs: 1.17–1.63, *p* = 0.00012, I^2^ = 0 (cohort *n* = 2, therefore I^2^ could be unstable); Figure 3E). A pooled estimate for the association between baseline ctDNA and PFS could not be calculated, because there were insufficient studies with HRs for this analysis to be pooled together.

Publication bias assessment was feasible only for the ctDNA decrease/clearance analysis for PFS (10 cohorts; Figure S1). Funnel-plot asymmetry suggested possible small-study effects, with trim-and-fill imputing 5 potentially missing studies and attenuating the pooled estimate from HR 0.34 (95% CI 0.25–0.47) to HR 0.45 (95% CI 0.32–0.64). However, the association remained statistically significant after adjustment. A publication bias assessment was not performed for the remaining pooled analyses because fewer than 10 cohorts were available.

**Figure 4:**
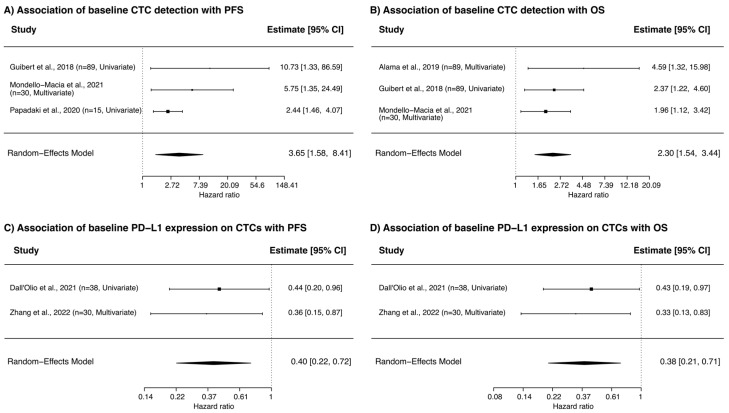
Forest plots of pooled hazard ratios (HRs) with 95% confidence intervals (CIs) for associations between baseline CTC-related biomarkers and clinical outcomes. (**A**) Baseline CTC detection and PFS [65,66,72]; (**B**) baseline CTC detection and OS [65,67,72]; (**C**) baseline PD-L1 expression on CTCs and PFS [60,63]; (**D**) baseline PD-L1 expression on CTCs and OS [60,63].

For CTCs, pooled analyses were feasible only for baseline CTC detection and baseline PD-L1 expression on CTCs, as these were the only constructs with sufficient comparability after harmonization of the effect direction and exclusion of non-comparable estimates. Baseline CTC detection was associated with worse clinical outcomes, with a pooled HR of 3.65 (95% CIs: 1.58–8.41, *p* = 0.002, I^2^ = 34.5) for PFS across three cohorts (patient *n* = 141; Figure 4A) and 2.30 (95% CIs: 1.54–3.44, *p* = 5.38 × 10^−5^, I^2^ = 0) for OS across three cohorts (patient *n* = 208; Figure 4B). Baseline PD-L1 expression on CTCs was associated with more favourable outcomes with pooled HRs of 0.40 (95% CIs: 0.22–0.72, *p* = 0.0023, I^2^ = 0) for PFS across two cohorts (patient *n* = 68; Figure 4C) and 0.38 (95% CIs: 0.21–0.71, *p* = 0.0021, I^2^ = 0) for OS (patient *n* = 68; Figure 4D). However, three other studies that could not be included in the pooled analysis did not show an association of PD-L1 status on CTCs with outcomes [59,65,72]. Although I^2^ was 0%, indicating no observed heterogeneity, this estimate should be interpreted cautiously because it was based on a small number of cohorts (*n* = 2–3) and may be unstable. These findings suggest that whilst the baseline CTC burden may have prognostic significance, the PD-L1 status on CTCs is an inconsistent biomarker. Overall, CTC biomarkers should be interpreted with caution given the small number of studies and substantial methodological heterogeneity in CTC-isolation platforms, detection thresholds, and PD-L1 scoring approaches.

### 3.5. Systematic Literature Analysis of Focus Themes

In addition to the pooled HR-based analyses, the full systematic review evidence base, comprising 28 ctDNA studies and 16 CTC studies, was assessed descriptively. Narrative synthesis was undertaken to retain important findings from studies that could not be included in the meta-analysis, such as those reporting non-poolable effect measures, differing analytical approaches, or other clinically relevant results. The descriptive assessment complements the quantitative synthesis and provides a complete overview of the available evidence. For clarity, non-pooled findings were summarized according to the following biomarker groups: baseline ctDNA burden, early on-treatment ctDNA decrease or clearance, ctDNA increase or persistence, bTMB, individual genomic alterations, baseline CTC burden, dynamic CTC changes, and PD-L1 expression on CTCs.

Across 28 ctDNA cohorts and 16 CTC cohorts, most studies sampled at baseline and again within the first two treatment cycles. At baseline, a higher ctDNA burden showed a prognostic association where outcomes were reported, relating to shorter PFS in three out of three cohorts and to shorter OS in two out of six cohorts, while several additional series described detectability without an explicit OS estimate. The consistent signal emerged on treatment: an early decline in ctDNA predicted a benefit in 17 of 18 cohorts for PFS and in 20 of 24 cohorts for OS, most often using a reduction of at least 50% or the clearance of tracked variants within the first two cycles. This association was evident from the earliest reassessment timepoints. At approximately three weeks, a benefit was observed in three of three cohorts for PFS and four of four cohorts for OS. At four to six weeks, a benefit was observed in seven out of seven cohorts for PFS and eight out of nine cohorts for OS. At seven to nine weeks, a benefit was observed in three out of three cohorts for PFS and four out of five cohorts for OS. When the first assessment occurred later or was not specified, the benefit remained in four out of five cohorts for PFS and four out of six for OS. Several cohorts reported that a ctDNA change had anticipated a radiographic response or progression by roughly one to two cycles.

Findings for bTMB and individual genomic alterations were heterogeneous across panels, and thresholds and were often underpowered.

For CTCs, detectable or higher baseline counts are generally tracked with inferior outcomes, relating to shorter PFS or OS in four out of five cohorts. Dynamic CTC changes showed a weaker and less consistent relationship than ctDNA, with some cohorts linking post-treatment decreases to improved outcomes but others reporting no association. A comprehensive description of the results from this systematic review is presented in the following paragraphs. Individual study details and outcome directions are presented in Table 1 and Table 2.

### 3.6. ctDNA Predictive Value in Immunotherapy Response

Studies presented in Table 1A evaluate the predictive value of ctDNA in immunotherapy-treated advanced NSCLC, using different analytical approaches and endpoints. ctDNA was assessed for detection and quantitatively as a measure of the tumour burden, including haploid genome equivalents per mL of plasma (hGE/mL), mean tumour molecules (MTM) per ml of plasma, maximum somatic allele frequency (MSAF), or tumour fraction (TFx), and as a genomic biomarker through the identification of specific variants, the variant/mutant allele frequency (VAF/MAF, %), defined as the proportion of ctDNA carrying specific tumour-derived variants/mutations relative to the total amount of circulating DNA in a blood sample, bTMB, and copy number changes. Likewise, the dynamic ctDNA response was defined heterogeneously across studies, including clearance to undetectable levels, percentage decrease from baseline, fold change between pre-treatment and on-treatment samples, or changes in allele frequency. Although methodological heterogeneity complicates direct comparisons across studies, several consistent signals emerge.

Overall, the evidence indicates that ctDNA provides prognostic and predictive information at both baseline and during immunotherapy treatment. Baseline ctDNA levels generally reflect the tumour burden and adverse disease biology, whereas early on-treatment ctDNA dynamics appear to identify benefit or resistance to ICIs earlier than conventional radiographic assessments. In addition, selected alterations detected in ctDNA, as well as ctDNA-derived mutational burden metrics, further refine outcome stratification.

Included ctDNA studies used both tumour-informed and tumour-agnostic approaches (Table 1). Tumour-informed assays relied on prior tumour genotyping to create patient-specific panels for tracking known variants over time, whereas tumour-agnostic assays used fixed plasma-based panels or broader sequencing approaches without prior tumour sequencing. In practice, this methodological distinction was closely linked to the assay breadth and analytical performance, with studies using approaches ranging from mutation-specific ddPCR or customised tracking panels to fixed targeted NGS panels, shallow whole-genome sequencing, and broader genomic profiling platforms. These differences likely contributed to variation in baseline detectability rates, thresholds for molecular responses, and the ctDNA metrics reported across studies.

### 3.7. Impact of Baseline ctDNA

**Figure 5:**
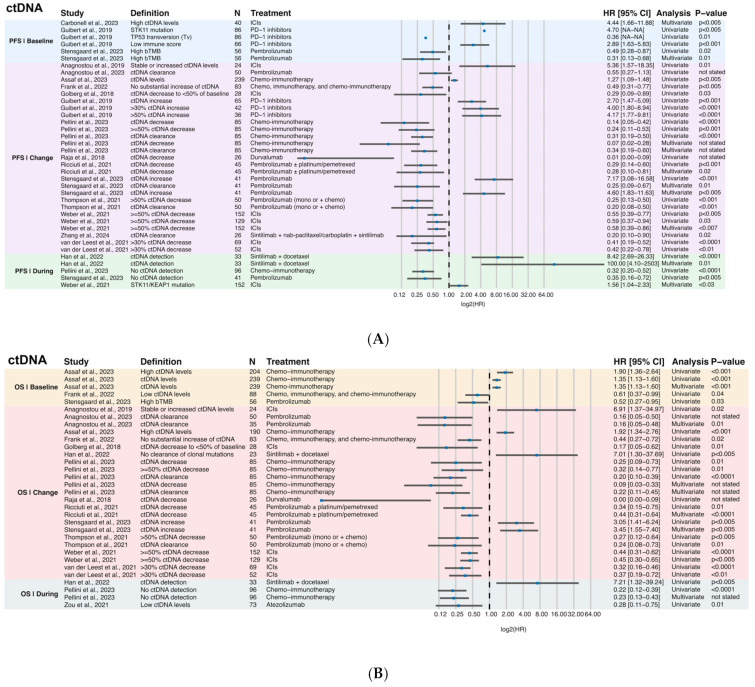
(**A**). Summary plot of reported hazard ratios (HRs) with 95% confidence intervals (CIs) for associations between ctDNA-based biomarkers and progression-free survival (PFS) across included studies of the narrative synthesis, grouped by baseline, change, and during-treatment assessments. The background colour shades indicate the main ctDNA analysis groups for PFS: light blue represents baseline analyses, light purple represents ctDNA change analyses, and light green represents during-treatment analyses [30,31,32,33,34,35,43,44,46,47,48,49,51,52,54,55]. (**B**). Summary plot of reported hazard ratios (HRs) with 95% confidence intervals (CIs) for associations between ctDNA-based biomarkers and overall survival (OS) across included studies of the narrative synthesis, grouped by baseline, change, and during-treatment assessments. The background colour shades indicate the main ctDNA analysis groups for OS: light yellow represents baseline analyses, light pink represents ctDNA change analyses, and light grey represents during-treatment analyses. The shading is used for visual separation of the analysis groups and does not indicate statistical significance, effect direction, study quality or weighting [30,31,32,33,34,35,43,44,46,47,48,49,51,53].

Across studies evaluating the baseline ctDNA status, undetectable or low pre-treatment ctDNA was consistently associated with more favourable outcomes, whereas detectable or higher baseline ctDNA was associated with worse survival and a lower probability of benefit from immunotherapy [30,32,41,42,43,52,54,56]. Baseline ctDNA was detectable in a large proportion of patients, although detection rates varied substantially by assay design and analytical approach, ranging from 47.5% (shallow whole-genome sequencing) to 96% in tumour-informed tracking studies [32,43,47,51,54,55]. Low baseline ctDNA was associated with improved OS (HR = 0.61), while high baseline ctDNA was associated with worse OS (HR = 1.90) and shorter PFS (HR = 4.44; Figure 5A,B) [32,33,54]. When analysed as a continuous variable, increasing baseline ctDNA levels also correlated with worse OS (HR = 1.35 in both univariate and multivariate analyses; Figure 5A,B) [32]. This adverse association of higher baseline ctDNA was consistent across different quantitative metrics, including MTM/mL, MSAF, TFx, and the SCNA burden, although different thresholds were used to define high ctDNA levels [32,41,54]. Higher baseline ctDNA was linked to shorter OS, poorer clinical outcomes, reduced likelihood of DCB, and lower probability of objective remission after treatment [32,41,42,43,56]. Baseline ctDNA also showed moderate-to-good discriminatory performance for non-response and death within two years of treatment initiation, with AUC values of 0.750 and 0.800, respectively [56]. In addition, higher TFx, particularly values ≥10%, and an increased SCNA burden were associated with more aggressive disease features, including bone and liver metastases, and with poorer outcomes based on ICI therapy [54].

### 3.8. Early On-Treatment ctDNA Dynamics

The most consistent finding across the included studies was that an early decline in ctDNA during treatment was associated with the radiographic response and longer survival, whereas persistent or increasing ctDNA identified patients with poor outcomes. This pattern was observed using different definitions of molecular response, including the percentage decline from baseline, clearance to undetectable ctDNA, and absence of an on-treatment increase [30,31,33,34,35,36,37,38,40,42,43,44,45,46,47,48,49,50,51,52,55,57].

When molecular response was defined as an early ctDNA decrease, studies consistently showed marked reductions in the risk of progression and death [34,43,44,45,48,49,51]. For example, a ctDNA decrease was associated with improved PFS and OS with HRs of 0.14% (Figure 5A) and 0.25 (Figure 5B), respectively, and these associations remained significant in multivariate analyses (PFS HR = 0.07; OS HR = 0.09; Figure 5A,B). In the same cohort, a ctDNA decrease was also associated with longer median PFS (8.8 vs. 3.5 months) and OS (not reached vs. 8.9 months) [43]. Similar findings were seen when response was defined as a ≥50% decline, with OS HRs ranging from 0.17 to 0.45 [34,43,48,51]. Corresponding survival differences included median PFS of 8.3 vs. 3.4 months and OS of 26.2 vs. 13.2 months [45] and PFS of 10 vs. 2 months and OS of 18.4 vs. 5.9 months [51]. A >30% decrease was also associated with improved outcomes, with PFS HRs of 0.41 and 0.42 and OS HRs of 0.32 and 0.37 [49]. Additional cohorts reported similar benefits with a ctDNA decrease overall, with PFS HRs of 0.29 and 0.28 and OS HRs of 0.34 and 0.44 in univariate and multivariate analyses, respectively (Figure 5A,B) [45].

A threshold of a 50% decline was the most frequently used and the most consistently associated with benefit. In the pooled analysis, a ≥50% decline was associated with improved PFS (HR = 0.34) and OS (HR = 0.36; Figure 3A,B). Similarly, across the narrative evidence base, an early ≥50% reduction in ctDNA was associated with radiographic tumour shrinkage and longer PFS and OS. These findings support ctDNA kinetics as an early marker of treatment efficacy. These associations were observed even in studies evaluating PD-L1 alongside ctDNA, suggesting that ctDNA dynamics add information beyond tissue-based biomarkers [34,38,42,43,48,50,51,57].

Comparable findings were observed when the molecular response was defined as ctDNA clearance, which was highlighted as particularly indicative of favourable treatment outcomes [30,31,43,47,48,52]. Clearance to undetectable ctDNA was associated with improved PFS and OS. Pooled analysis resulted in PFS HR = 0.32. Reported HRs from the narrative synthesis were 0.20–0.55 for PFS and 0.16–0.24 for OS (Figure 5A,B) [30,43,47,48,52]. ctDNA clearance after two cycles was also associated with prolonged PFS of 18.1 vs. 4.3 months [52]. ctDNA response for predicting the RECIST response had a sensitivity of 82% and a specificity of 75%, demonstrating strong concordance between ctDNA molecular responses and traditional radiographic assessment [30]. Importantly, ctDNA clearance also identified favourable outcomes among patients with radiological stable disease, indicating that the molecular response may capture benefits earlier than imaging alone [31,52].

Several studies also showed that ctDNA changes preceded conventional imaging. A molecular response was detected at a median of 24.5 days compared to 72.5 days via radiographic assessment. ctDNA anticipated radiological progression by a median of 1.5 months in most progressing patients, while clonal progression was detectable 2–4 months before radiographic progression in some cohorts [33,34,41]. Reductions at six weeks post-treatment not only predicted prolonged survival but also correlated with significant reductions in tumour volume, often preceding radiographic responses [44].

By contrast, persistent ctDNA detection or rising ctDNA during treatment consistently identified poor prognosis [30,32,33,35,47]. Stable or increased ctDNA was associated with substantially shorter survival, with HRs of 5.36 for PFS (Figure 5A) and 6.91 for OS (Figure 5B) [30]. A ctDNA increase during treatment was similarly associated with worse PFS and OS. Reported HRs ranged from 2.70 to 7.17 for PFS and from 3.05 to 3.45 for OS (Figure 5A,B), depending on the threshold and model used [47,51]. This was also reflected in absolute survival differences, including median PFS of 2.19 vs. 11.21 months and OS of 7.78 vs. 24.20 months [47], as well as PFS of 2 vs. 14 months [51]. Persistent ctDNA detection during treatment was also associated with poor outcomes, including PFS HRs of 8.42 and 100.0 and an OS HR of 7.21 (Figure 5A,B) [35]. Conversely, the absence of a substantial ctDNA increase was associated with improved survival, with HRs of 0.49 for PFS and 0.44 for OS (Figure 5A,B) [33].

### 3.9. Critical ctDNA Testing Timepoints

Studies assessing ctDNA at predefined early landmarks showed that clinically informative changes can be detected within the first weeks of treatment, often before the first radiological assessment. Overall, the most informative window for ctDNA monitoring appeared to be between 2 and 9 weeks after treatment initiation.

At around 2 weeks, a substantial decrease in ctDNA AF was already associated with a later radiographic response and DCB [36,37,46,55]. Within the first 4–6 weeks, a marked decline in ctDNA was repeatedly associated with improved PFS and OS [34,42,47,51,55]. For example, a ctDNA reduction to <50% of baseline was associated with a PFS HR of 0.29 (Figure 5A) and an OS HR of 0.17 (Figure 5B) [34]. Similarly, >30% ctDNA reduction at 4–6 weeks was associated with PFS HRs of 0.41 and 0.42 and OS HRs of 0.32 and 0.37 [49]. The absence of ctDNA detection after one or two cycles was likewise associated with favourable outcomes, including a PFS HR of 0.35 (Figure 5A) [47].

The 6-week timepoint was the most consistently evaluated timepoint, when lower ctDNA levels, ctDNA clearance, or a substantial decline from baseline were all associated with longer survival and better treatment response [30,32,35,44,45,47,50,52,53]. Quantitatively, lower on-treatment ctDNA was associated with improved OS (HR = 0.28; Figure 5B), while persistently high on-treatment ctDNA was associated with worse OS (HR = 1.92; Figure 5B), and rising ctDNA was associated with shorter PFS (HR = 1.27; Figure 5A) [32,53]. These molecular changes often preceded radiographic findings and improved risk stratification beyond RECIST alone [32,44].

At later timepoints, including 8–9 weeks, the same overall pattern was maintained [31,38,43,48,57]. ctDNA reductions from baseline to these timepoints were strongly associated with favourable PFS and OS, particularly when defined as a ≥50% decrease or complete clearance [31,43,48]. Reported effect sizes included PFS HRs of 0.24–0.25 and OS HRs of 0.27–0.32 for a ≥50% decrease, and PFS HRs of 0.20–0.31 and OS HRs of 0.20–0.24 for ctDNA clearance (Figure 5A,B) [43,48]. Around 9 weeks, the conversion to undetectable ctDNA was especially characteristic of patients with better outcomes, whereas non-responders generally showed little or no meaningful ctDNA change clearance [31,43,48].

### 3.10. Impact of Specific Mutations

Several studies also evaluated whether specific genomic alterations detected in ctDNA were associated with the response or resistance to immunotherapy [35,38,39,41,51,52]. Although these findings were less uniform than those relating to ctDNA kinetics, they collectively suggest that ctDNA can capture both baseline resistance biology and molecular evolution under treatment pressure.

At baseline, alterations in genes linked to tumour-suppressor functions or immune resistance were generally associated with poorer outcomes. Mutations in *BRCA2*, *BRINP3*, *FBXW7*, *KIT*, *RB1*, *PTEN*, *STK11*, and *KEAP1* were associated with shorter PFS or early progression [51,52]. Where quantified, *STK11* mutations were associated with worse PFS (HR = 4.70; Figure 5A), and *STK11*/*KEAP1* mutations remained independently associated with poorer PFS during treatment (HR 1.56; Figure 5A) [51]. By contrast, transversion mutations in *KRAS* or *TP53* were associated with more favourable outcomes in this cohort, with *TP53* transversion significantly associated with improved PFS (HR = 0.36) and *KRAS* transversion showing a similar trend (HR = 0.46; Figure 5A). A composite “immune score” was also proposed, in which a high score was defined by the absence of targetable drivers, *PTEN*, or *STK11* alterations together with the presence of *KRAS* or *TP53* transversion mutations; this was associated with improved outcomes, with median PFS of 14 months versus 2 months for the low-score group (HR = 2.89; Figure 5A) [55]. Additional mutational analyses suggested the differential enrichment of specific alterations in responders compared to non-responders. Alterations in *PIK3CG* and *RBM10* were enriched among responders, whereas *EGFR*, *DNMT3A*, *ABL2*, *MAGI2*, and *EPHA7* were associated with non-response or progression [35].

At progression, ctDNA profiling also provided insight into acquired resistance [38,39,41]. Emerging alterations involved pathways associated with immune escape and tumour adaptation, including Wnt-pathway-related genes, increased copy number aberrations, tumour-suppressor loss such as *PTEN*, and alterations in immune-related genes including *B2M* [39,41]. ctDNA analysis of neoantigen evolution further suggested that acquired resistance may arise through immunoediting and clonal selection under treatment pressure, allowing the longitudinal tracking of whether neoantigen-bearing clones persist, become lost, or are replaced during immunotherapy [38].

### 3.11. Blood Tumour Mutational Burden

Studies included in this analysis have investigated the role of bTMB, measured as the number of mutations per megabase (Mb), as a predictive biomarker of immunotherapy response. Overall, higher bTMB was generally associated with more favourable outcomes, although the evidence base was smaller and less standardized than that for ctDNA kinetics [39,41,42,47,50]. It was shown that a high bTMB was associated with improved survival, with PFS and OS HRs of 0.49 (Figure 5A) and 0.52 (Figure 5B), respectively, in univariate analysis and a multivariate PFS HR of 0.31 (Figure 5A) [47]. In addition, ctDNA-normalized bTMB was associated with improved clinical outcomes, suggesting that adjusting bTMB for the ctDNA concentration may improve its interpretability by reflecting the mutational load relative to the tumour-derived DNA burden rather than absolute mutation counts alone. Combining the ctDNA-normalized bTMB with tumour PD-L1 expression and circulating CD8+ T-cell levels further improved the classification of clinical benefit, supporting the value of multi-parameter biomarker models [42]. In contrast, the bTMB measured at progression did not show a significant change from baseline, suggesting that the dynamic bTMB may be less informative than early ctDNA quantity changes for monitoring the treatment response [39].

### 3.12. CTCs’ Predictive Value in Immunotherapy Response

Studies reviewed in Table 2 explore the predictive value of CTCs and their PD-L1 expression in determining the response to immunotherapy in advanced NSCLC patients. Each study primarily focuses on isolating and quantifying CTCs through different methods, evaluating PD-L1 expression on CTCs either at baseline or after treatment, and assessing the correlation with PFS and OS. While common trends emerge, significant variability in findings exists, particularly in how CTC counts and PD-L1 expression on these cells relate to treatment outcomes.

### 3.13. CTC Counts at Baseline

**Figure 6:**
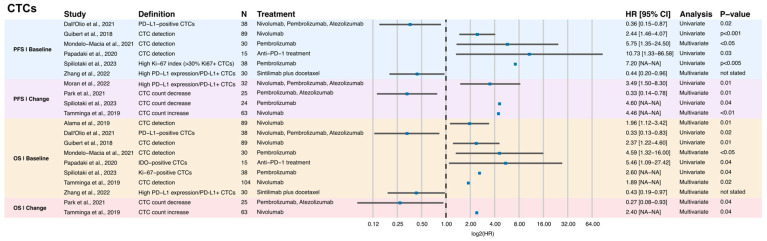
Summary plot of reported hazard ratios (HRs) with 95% confidence intervals (CIs) for associations between CTC-based biomarkers and progression-free survival (PFS) and overall survival (OS) across included studies of the narrative synthesis, grouped by baseline and change assessments. The background colour shades indicate the four main CTC analysis groups: light blue represents PFS baseline analyses, light purple represents PFS change analyses, light yellow represents the OS baseline analyses, and light pink represents OS change analyses. The shading is used for visual separation of these groups and does not indicate statistical significance, effect direction, study quality or weighting [58,59,60,61,63,65,66,67,68,72].

Accumulating evidence across studies employing diverse CTC isolation platforms indicates that both the presence of CTCs and elevated baseline CTC counts are consistently associated with inferior clinical outcomes in metastatic NSCLC patients receiving immune checkpoint inhibitors (ICIs), while further molecular characterization may enhance their predictive and prognostic utility. Studies evaluating both the predictive and prognostic value of CTC detection in NSCLC patients treated with ICIs have generally included relatively small to moderate cohorts (15–104 patients) and consistently demonstrate an association among CTC presence, elevated baseline CTC counts, and poorer clinical outcomes (Figure 4A,B). Across different CTC isolation platforms, including CellSearch, ISET and Parsortix, the presence of detectable CTCs prior to treatment initiation was associated with significantly shorter PFS, with reported HRs of 5.7 and 10.7 (Figure 6) [65,66]. Analysis using the CellSearch platform demonstrated that the presence of CTCs both at baseline and during treatment was significantly associated with inferior OS (HR = 1.89; Figure 6) [68].

Similarly, high baseline CTC counts (>30 CTCs/7.5 mL), assessed using the ISET platform, were associated with worse PFS (HR = 2.44) [72], whereas analysis using the ScreenCell filtration-based method demonstrated that lower baseline CTC counts (<2 cells) were associated with longer survival compared with higher counts (HR = 1.96; Figure 6) [67], further supporting the role of the pre-treatment CTC burden as a marker of poor clinical outcomes. Additional molecular characterization strengthened this association, as a high proliferative index (>30% Ki-67-positive CTCs) was linked to shorter PFS (HR = 7.2; Figure 6) [58]. Moreover, the detection of indoleamine-2,3-dioxygenase (IDO)-positive CTCs at baseline was associated with shorter OS (HR = 5.4; Figure 6) [66], highlighting the added prognostic value of immune-related CTC characterization.

### 3.14. PD-L1 Expression on CTCs

While CTC enumeration is a commonly used biomarker, the role of programmed death-ligand 1 (PD-L1) expression on CTCs in predicting response to immunotherapy remains complex and inconsistent across studies, whilst most studies include relatively small cohorts (30–89 patients). Some studies suggest that higher PD-L1 expression on CTCs is associated with improved clinical outcomes, as shown based on the quantitative analysis (Figure 4C,D). For example, patients with >50% PD-L1-positive CTCs demonstrated improved responses to immunotherapy (three of four patients) [71], and elevated PD-L1 expression (≥32.5% PD-L1-positive CTCs) was associated with significantly improved PFS and OS (HR: 0.44 and 0.43, respectively; Figure 6) [60]. Furthermore, PD-L1 expression on CTCs has been associated with improved PFS and OS (HR: 0.36 and 0.33, respectively; Figure 6), whereas patients lacking PD-L1 expression on CTCs exhibited poorer clinical outcomes [63]. However, three other studies have not identified a significant association between PD-L1 expression on CTCs and patient prognosis [59,65,72], and, as a result, PD-L1 status on CTCs should be considered an inconsistent biomarker.

Of note, PD-L1-positive CTCs have been detected even in patients with PD-L1-negative primary tumours, without a good correlation in other studies [69]. Beyond PD-L1 expression alone, the additional characterization of CTCs may further enhance their clinical relevance; for instance, the presence of Ki-67-positive CTCs was associated with worse OS (HR = 2.6; Figure 6) [58].

### 3.15. Dynamic CTC Changes and PD-L1 Expression

Several studies have investigated the dynamic monitoring of CTC counts and PD-L1 expression during treatment, suggesting that the longitudinal assessment of these biomarkers may provide additional predictive and prognostic information for patients’ ICIs [64,70,73]. However, only a limited number of studies have specifically evaluated the clinical relevance of dynamic changes in CTC counts and PD-L1 expression in relation to the treatment response.

In one study involving 25 patients, the CD-PRIME™ system was used to monitor CTC dynamics during ICI therapy. A reduction in CTC counts after the first treatment cycle was significantly associated with improved PFS and OS (HRs: 0.33 and 0.27, respectively; Figure 6) [61]. Similarly, a decrease in total CTC counts and PD-L1–low CTCs after the first cycle of pembrolizumab was associated with a longer PFS compared with patients showing increased CTC counts, indicating that early changes in the CTC burden and PD-L1 expression may serve as indicators of the treatment response [58]. Another study showed that an increase in multiploid PD-L1-positive circulating tumour endothelial cells (CTECs) during treatment was observed in patients with progressive disease and was associated with shorter PFS [69]. Conversely, an increase in PD-L1 expression on tumour-associated cells (TACs), including CTCs and cancer-associated macrophage-like cells (CAMLs), from low to high levels after treatment was significantly associated with improved PFS (HR = 3.49; Figure 6) [59]. In addition, patients with ≥7.7% PD-L1-positive CTCs at eight weeks exhibited significantly longer PFS compared with those with lower PD-L1 expression levels [62].

## 4. Discussion

### 4.1. Summary of Main Findings

This systematic review and meta-analysis support the potential role of liquid biopsy, using ctDNA and CTC analysis, as promising minimally invasive biomarkers for predicting the immunotherapy response in advanced NSCLC. A higher baseline ctDNA burden and detectable or elevated baseline CTC counts generally tracked with poorer outcomes, whereas an early on-treatment ctDNA decline repeatedly correlated with the treatment benefit. The observed associations are biologically plausible because ctDNA reflects tumour-derived DNA released into the circulation from dying or actively turning-over tumour cells. In patients responding to ICIs, effective anti-tumour immune activation may reduce the number of viable tumour cells and overall tumour burden, thereby decreasing DNA shedding and leading to an early ctDNA decline or clearance. Conversely, persistent or increasing ctDNA during treatment may indicate ongoing tumour growth, incomplete immune-mediated tumour control, or resistant tumour clones and, therefore, poorer outcomes [4,34]. As molecular changes are often preceding radiographic changes, ctDNA dynamics may provide earlier indications of the treatment response. Likewise, detectable baseline CTCs may reflect greater tumour dissemination and metastatic potential and are therefore associated with poorer outcomes [67,68]. The most reproducible ctDNA signal across studies was an early decrease in ctDNA, commonly defined as a reduction of at least 50% from baseline or clearance to undetectable levels within the first two to three treatment cycles. Several cohorts also suggested that ctDNA dynamics may precede the radiographic response or progression by one or more treatment cycles, supporting the biological and clinical relevance of early molecular monitoring. By contrast, findings for the bTMB, individual genomic alterations, PD-L1 expression on CTCs, and dynamic CTC phenotypes were more heterogeneous and less consistently replicated.

The meta-analysis strengthened these narrative observations by showing that early on-treatment ctDNA kinetics were the most robust and comparable liquid-biopsy signal across studies. Pooled analyses demonstrated that a ctDNA decrease/clearance was associated with significantly improved PFS and OS, whereas a ctDNA increase/persistence was associated with worse outcomes. Exploratory subgroup analyses showed that this favourable association remained directionally consistent when the molecular response was defined more specifically as a ≥50% ctDNA decrease or ctDNA clearance, although the OS clearance subgroup was less precise because of smaller numbers. An exploratory pooled analysis also suggested that higher baseline ctDNA was associated with a worse OS, but baseline ctDNA constructs overall were less standardized and less comparable than on-treatment kinetics. Together, these findings support early ctDNA dynamics as the most reproducible liquid-biopsy correlate of outcome in this setting.

For CTCs, the narrative synthesis suggested that the baseline CTC burden is more consistently informative than dynamic CTC changes, while the pooled analyses supported an association between baseline CTC detection and worse PFS and OS. Baseline PD-L1 expression on CTCs was associated with more favourable outcomes in the pooled analysis but was not associated with better outcomes in three other studies, hence this finding should be interpreted cautiously because of the small evidence base and substantial methodological heterogeneity across CTC enrichment methods, phenotypic definitions, and PD-L1 scoring strategies.

Overall, the current evidence suggests that baseline ctDNA levels and ctDNA kinetics are the most reproducible liquid-biopsy marker associated with outcomes in advanced NSCLC treated with ICIs, whereas CTC-based biomarkers remain promising but less mature. Our findings are broadly consistent with previous reviews and meta-analyses evaluating liquid biopsy biomarkers in NSCLC, although the clinical setting, treatment context, and biomarker definitions differed across studies [15,16,17,18,19,20,22,23,24,25,26]. Specifically, Zhong et al. demonstrated that ctDNA-based MRD detection after definitive therapy predicts relapse and survival in lung cancer [16], while Fan et al. reported prognostic associations for ctDNA-detected EGFR and KRAS mutations in advanced NSCLC, mainly in targeted therapy or chemotherapy settings [18]. Moreover, Wang et al. confirmed the predictive value of on-treatment ctDNA reductions in patients receiving immunotherapy; however, they did not identify a significant prognostic association for baseline ctDNA [17]. Collectively, these studies support the broader clinical relevance of ctDNA- and CTC-based biomarkers in NSCLC, but do not fully address the predictive value of both ctDNA and CTCs in patients receiving ICIs. In contrast, the present study specifically evaluated patients with advanced NSCLC receiving ICIs and quantitatively synthesized the available evidence for both ctDNA and CTC biomarkers with a particular focus on baseline ctDNA/CTC burden and early-on treatment dynamics. The pooled analyses strengthen previous observations by demonstrating that an early-on treatment ctDNA decline or clearance represents the most consistent liquid-biopsy marker associated with improved survival outcomes in this clinical setting.

### 4.2. Limitations of the Evidence

Despite these promising findings, the overall evidence base remains limited by small cohort sizes, variability in treatment regimens and patient characteristics, inconsistent timing of blood sampling, and substantial methodological heterogeneity in both ctDNA and CTC analyses. These factors may directly influence biomarker measurements, including the ctDNA concentration, sensitivity of mutation detection and quantification, CTC counts, and PD-L1 expression on CTCs, thereby complicating interpretations across studies.

For ctDNA, different next-generation sequencing assays were used for detection and quantification, with important variation in the panel design, analytical sensitivity, specificity, and detection limits. Such differences are likely to affect both the identification of ctDNA-derived alterations and the magnitude of measured ctDNA changes, adding complexity to cross-study comparisons and limiting immediate clinical translation.

For CTCs, even greater heterogeneity arose from the use of multiple isolation and characterization platforms, including CellSearch, Parsortix, ISET, and ScreenCell, each of which relies on different biological or physical principles and may capture different CTC subpopulations. This is particularly relevant for analyses of CTC enumeration and PD-L1 expression, where platform-dependent differences in enrichment and detection may contribute substantially to discordant findings across studies.

The broader narrative evidence base was therefore considerably wider than the subset of studies eligible for pooling, and the meta-analysis necessarily reflects the most clinically and methodologically comparable constructs rather than the full spectrum of reported liquid-biopsy biomarkers. Lastly, publication bias could be explored formally only for the largest pooled ctDNA contrast, and trim-and-fill suggested that the protective effect for ctDNA decrease/clearance and PFS may be somewhat overestimated by small-study effects, although the adjusted association remained statistically significant.

### 4.3. Limitations of the Review Process

No protocol was prospectively registered; however, eligibility criteria, outcomes, data items, and synthesis methods were defined before quantitative pooling and are reported in detail. Heterogeneity in effect measures, assay classes, thresholds, and sampling timepoints limited quantitative synthesis for several constructs; however, pooled analyses were feasible for selected ctDNA and CTC biomarker groups after harmonization of effect direction and the exclusion of non-comparable or non-independent estimates. Although structured quality and risk-of-bias assessments were performed, substantial methodological diversity across studies reduced comparability and limited certainty for several pooled and narrative findings. Greater standardization of assay methodology, sampling timepoints, response thresholds, and reporting practices will be essential to strengthen future evidence synthesis in this field.

### 4.4. Implications for Clinical Practice

There is an unmet clinical need to implement real-time minimally invasive molecular analyses to capture the therapeutic response and detect early disease relapse to help decision-making in the context of precision immunotherapy. The ability to monitor ctDNA and CTCs non-invasively through blood samples rather than invasive tissue biopsies offers a safer and earlier alternative for patients, reducing the risks associated with traditional biopsy methods. ctDNA and CTCs, not only supplement the information obtained from tissue biopsies but, in some cases, may offer advantages over them.

From a clinical perspective, liquid biopsy should currently be considered complementary to radiological assessments rather than a replacement for it. In particular, an early ctDNA decline or clearance may help identify treatment benefits before the first routine imaging assessment, whereas persistent or increasing ctDNA levels may indicate a higher likelihood of early treatment resistance and warrant closer monitoring. Baseline CTC detection may provide additional prognostic stratification; however, current evidence does not support treatment decisions based on CTC phenotypes such as PD-L1 expression. Consequently, treatment decisions should not yet be based solely on ctDNA or CTC findings, as clinically validated thresholds and standardized testing protocols remain lacking. Therefore, these biomarkers should currently be viewed as promising adjunctive tools requiring further validation in prospective studies.

### 4.5. Implications for Research and Future Directions

Moving forward, standardization and validation should be the main priority for the clinical adoption of ctDNA and CTC analysis. To incorporate ctDNA assays into the management of advanced NSCLC treated with ICIs or chemo-immunotherapy, assay-specific thresholds for analytical sensitivity, including the limit of detection, and specificity must be clearly defined. Future studies should therefore prioritize assay standardization, prespecified ctDNA response thresholds, and harmonized early sampling landmarks, particularly within the first 2 to 6 weeks of treatment, to improve comparability across cohorts and enable more informative pooled analyses. Clinical utility must then be demonstrated in prospective trials evaluating whether baseline and dynamic ctDNA changes can improve treatment monitoring and decision-making beyond current radiological assessment.

Beyond conventional mutation-based ctDNA testing, emerging liquid biopsy approaches now integrate non-genomic cfDNA features, such as fragmentation patterns and methylation signatures, using machine learning to enhance predictive accuracy [74,75,76,77]. Importantly, integrating methylation and fragmentomic data has also shown predictive value in the pembrolizumab response, independent of the tumour type, PD-L1 status, or TMB [5]. Additionally, the use of artificial intelligence and machine learning to analyse large datasets generated from ctDNA and CTC analysis could help identify novel predictive signatures that might be missed using traditional methods [78]. This integrative technological approach has the potential to markedly improve the precision and effectiveness of liquid biopsy biomarker-driven treatment strategies.

Future trials and prospective studies should also investigate multimodal biomarker strategies combining ctDNA dynamics with established biomarkers [79] such as tumour PD-L1 expression, the blood or tissue mutational burden, radiomic features, and immune-related circulating biomarkers to improve predictive accuracy and refine patient selection for immunotherapy.

## 5. Conclusions

This systematic review supports the clinical relevance of liquid biopsy, particularly ctDNA and CTC analysis, in advanced NSCLC treated with immunotherapy. Across the narrative synthesis, a higher baseline ctDNA burden and detectable or elevated baseline CTC counts were generally associated with poorer outcomes, while an early on-treatment ctDNA decline or clearance repeatedly correlated with longer PFS, longer OS, and radiological benefits. The meta-analysis strengthened these observations by showing that an early on-treatment ctDNA decrease/clearance was the most consistent pooled liquid-biopsy signal associated with improved outcomes, whereas a ctDNA increase/persistence was associated with worse prognosis. Exploratory pooled analyses further supported the relevance of a ≥50% ctDNA decrease and ctDNA clearance, while the baseline ctDNA level showed a weaker and less standardized adverse association limited to OS. For CTCs, pooled analyses supported the adverse prognostic significance for baseline CTC detection and favourable prognostic significance for positive PD-L1 expression on CTCs, whilst the evidence for dynamic CTC changes remained limited and methodologically heterogeneous. Taken together, these findings suggest that liquid biopsy, whilst promising, should be considered an adjunct tool for early-response monitoring and prognostic stratification rather than as a replacement for radiologic assessments. At present, ctDNA and CTC monitoring may complement, but cannot replace, radiological assessments in guiding immunotherapy decisions in patients with advanced NSCLC; therefore, future prospective trials are needed, which will incorporate serial ctDNA and CTCs using standardized assays, harmonized early sampling timepoints, and pre-specified molecular-response thresholds to confirm its clinical utility.

## Figures and Tables

**Table 1:** (**A**). Studies that examined ctDNA as a response biomarker in advanced NSCLC treated with immunotherapy, following a tumour-informed approach. (**B**). Studies that examined ctDNA as a response biomarker in advanced NSCLC treated with immunotherapy, following a tumour-agnostic approach. (**C**) A single study that examined ctDNA as a response biomarker in advanced NSCLC treated with immunotherapy, following both a tumour-informed and tumour-agnostic approach. Progression-free survival (PFS), overall survival (OS), response rate (RR), limit of detection (LOD), Level of Evidence (LoE), Not Reported (NR) and REMARK (RE).

(**A**)										
**Study** **Pubmed ID**	**Sample size**	**Assay**	**No of genes**	**LOD**		**LoE**	**RE**	**Treatment**	**Result: Baseline ctDNA**	**Result: Change in ctDNA**
Anagnostou et al., 2023 [30] PMID: 37814061	50	Custom ctDNA NGS assay	NR	NR		V	19	pembrolizumab	Baseline maxMAF not predictive of response. Patients with undetectable baseline ctDNA had longer PFS/OS.	Greater ctDNA reduction after cycles 2 and 3 predicted RECIST response and longer survival. ctDNA molecular response, defined as maximal mutant allele fraction clearance, demonstrated high sensitivity and specificity for predicting radiographic RECIST response and was associated with significantly improved PFS and OS.
Anagnostou et al., 2019 [31] PMID: 30541742	24	TEC-Seq (Targeted Error Correction Sequencing)	58	~0.1%		V	19	immune checkpoint blockade		ctDNA decrease at early treatment timepoints (within 4–8 weeks) correlated with response.
Assaf et al., 2023 [32] PMID: 36928816	466	FoundationOne Liquid CDx + custom panel	311	~0.1%		IV	20	chemo-immunotherapy	High ctDNA at baseline was linked to poor OS.	Changes in ctDNA levels after cycles 2 and 3 are strongly associated with OS. A reduction in ctDNA at these timepoints correlated with treatment response. Early testing of ctDNA is more predictive of trial outcomes than early radiographic imaging.
Frank et al., 2022 [33] PMID: 36969747	132	Custom droplet digital PCR	Mutation-specific	Low		V	16	chemotherapy, immunotherapy or combination treatment		ctDNA rise anticipated radiologic progression. ctDNA monitoring allowed for earlier detection of treatment failure compared to radiologic assessments.
Goldberg et al., 2018 [34] PMID: 29330207	28	Error-suppressed deep sequencing	24 (43 mutation hotspots)	NR		V	18	immune checkpoint inhibitors		A >50% drop in mutant allele fraction predicted longer PFS and OS. ctDNA response preceded to radiological response (RR).
Han et al., 2022 [35] PMID: 36600554	33	Targeted NGS	448	NR		V	18	sintilimab plus docetaxel		ctDNA clearance at week 6 linked to decreased tumour volume and significantly longer PFS and OS. Residual ctDNA was an independent risk factor for poor prognosis.
Horndalsveen et al., 2023 [36] PMID: 36330681	21	Custom ctDNA NGS assay	NR	NR		V	14	atezolizumab combined with radiotherapy		Rapid ctDNA drop to <30% of baseline linked with response or prolonged benefit.
Iijima et al., 2017 [37] PMID: 29078173	14	Custom PCR + Sequencing	Mutation-specific	NR		V	15	nivolumab		Decrease in ctDNA allele frequency at 2 weeks showed 100% concordance with treatment response.
Jia et al., 2020 [38] PMID: 32382482	10	Customized panels to each individual	18–30	NR		V	18	immune checkpoint blockade		>50% ctDNA drop at 8 weeks correlated with longer PFS. Early ctDNA decline also correlated with tumour shrinkage.
Leprieur et al., 2020 [39] PMID: 32581058	8	Whole-exome sequencing	Exome-wide	NR		V	15	immune checkpoint inhibitors		Clonal evolution at 6 months after initial response and resistance mechanisms (e.g., PTEN loss, Wnt pathway alterations) seen in progression.
Leprieur et al., 2018 [40] PMID: 29721388	20	Not specified	NR	NR		V	16	nivolumab		Increase >9% in ctDNA at ~2 months predicted poor response. Stable or decreasing ctDNA indicated benefit.
Li et al., 2019 [41] PMID: 31692284	12	Ultra-deep sequencing (Originated 329 panel)	329	NR		V	16	anti-PD1 treatment	MSAF levels reflected tumour burden.	Decline in MSAF after 2 cycles predicted response. Clonal progression identified 2–4 months prior to radiographic progression.
Nabet et al., 2020 [42] PMID: 33007267	99	DIREct-On model + custom sequencing panel	NR	NR		V	19	PD-L1 blockade alone or in combination with CTLA-4 blockade or chemotherapy	Pre-treatment ctDNA levels and peripheral CD8 T-cell counts were independently associated with durable clinical benefit (DCB).	Early ctDNA reduction at day 21 predicted DCB and outperformed individual features when combined with PD-L1 expression and CD8+ T cell levels.
Pellini et al., 2023 [43] PMID: 37702716	133	FoundationOne Tracker	Up to 16 variants from >300-gene CGP panel	NR		V	18	chemo-immunotherapy	High baseline ctDNA correlated with poor prognosis.	ctDNA decrease from baseline to cycle 4 associated with longer PFS and OS.
Raja et al., 2018 [44] PMID: 30093454	100	Custom assay	73	NR		V	18	durvalumab		Early reduction in VAF at 6 weeks correlated with decreased tumour volume and significantly better PFS and OS. The ctDNA reduction often preceded RR.
Ren et al., 2022 [45] PMID: 34923163	193	Custom assay	NR	NR		V	14	camrelizumab plus chemotherapy		ctDNA clearance after 2 cycles associated with longer PFS and OS.
Ricciuti et al., 2021 [46] PMID: 33771889	62	Enhanced tagged-amplicon sequencing	36	NR		V	18	pembrolizumab		ctDNA decrease at 21 days correlated with RR and longer PFS and OS.
Stensgaard et al., 2023 [47] PMID: 37323168	56	AVENIO ctDNA Surveillance Kit (Roche)	197	~0.1%		V	18	pembrolizumab	High blood TMB at baseline linked to improved survival.	Increased ctDNA levels after 1–2 cycles correlated with worse PFS and OS.
Thompson et al., 2021 [48] PMID: 34095713	67	Guardant360 and OMNI	74 (Guardant360)/500 (OMNI)	~0.1%		V	17	pembrolizumab		>50% decrease in ctDNA at 9 weeks predicted longer PFS and OS ctDNA response correlated with RR.
Van der Leest et al., 2021 [49] PMID: 34449963	100	ddPCR	Mutation-specific	0.01–0.1%		V	16	immune checkpoint inhibitors		>30% decrease in ctDNA at 4–6 weeks linked with higher DCB, prolonged PFS, and OS.
Wang et al., 2022 [50] PMID: 35197718	9	Targeted NGS	NR	NR		V	14	pembrolizumab		ctDNA reduction at week 3, 6 correlated with DCB. ctDNA increase at week 6 correlated with non-DCB. ctDNA dynamics linked to PFS/OS.
Weber et al., 2021 [51] PMID: 34994642	177	AVENIO ctDNA Expanded Kit (Roche)	77	~0.1%		V	19	immune checkpoint inhibitors		≥50% decrease in ctDNA at ~4–6 weeks associated with improved PFS and OS.
Zhang et al., 2024 [52] PMID: 38374204	47	NGS	NR	NR		V	16	sintilimab	Negative ctDNA at baseline associated with longer PFS.	ctDNA clearance after 2 cycles associated with improved PFS.
Zou et al., 2021 [53] PMID: 34994614	94	AVENIO ctDNA Surveillance Kit (Roche)	197	~0.1%		V	19	atezolizumab/docetaxel	High baseline ctDNA levels (mutant molecules per mL; MMPM) linked to OS.	Median MMPM at 6 weeks significantly associated with OS Low ctDNA was associated with better survival.
(**B**)										
**Study** **Pubmed ID**	**Sample size**	**Assay**	**No of genes**	**LOD**	**LoE**	**RE**	**Treatment**	**Result: Baseline ctDNA**	**Result: Change in ctDNA**	
Carbonell et al., 2023 [54] PMID: 36852704	45	Shallow whole-genome sequencing (sWGS)	Genome-wide	NR	V	17	immune checkpoint inhibitors	Tumour Fraction (TFx) ≥10% was associated with shorter PFS.	On-treatment ctDNA at 2–3 weeks better predicted efficacy than baseline TFx alone.	
Guibert et al., 2019 [55] PMID: 31518912	97	Inivata amplicon sequencing	36	NR	V	17	PD-L1 inhibitors	PTEN/STK11 mutations associated with early progression.	Decrease in allele fraction at 1 month predicted better PFS.	
Wang et al., 2023 [56] PMID: 37324075	143	qpCR-based ctDNA quantification	Not-panel based	NR	V	19	immune checkpoint inhibitors	ctDNA < 3.72 ng/μL before treatment predicted objective response (CR/PR).	Lower ctDNA at cycle 4 associated with improved outcomes.	
(**C**)										
**Study** **Pubmed ID**	**Sample size**	**Assay**	**No of genes**	**LOD**	**LoE**	**RE**	**Treatment**	**Result: Baseline ctDNA**	**Result: Change in ctDNA**	
Cheng et al., 2023 [57] PMID: 37184093	19	Custom tumour-informed NGS 671- gene panel (OriMIRACLE S™)	671 (tumour-agnostic)	NR	V	16	first-line ICI monotherapy or combined with chemotherapy	N/A	Decrease in ctDNA burden predicted PR. ctDNA drop preceded RR in most patients.	

**Table 2:** Included studies that examined CTCs as a response biomarker in advanced NSCLC treated with immunotherapy. Progression-free survival (PFS), overall survival (OS), response rate (RR), Level of Evidence (LoE), REMARK (RE).

Study PubMed ID	Sample Size	Method	Biomarker	LoE	RE	Treatment	Result: Baseline	Result: CTCs Change
Spiliotaki et al., 2023 [58] PMID:36177552	47	IF staining (Ficoll)	CTCs, PD-L1, Ki-67	V	15	pembrolizumab	A high Ki-67 index (>30% Ki67 + CTCs) prior to treatment was associated with shorter PFS. Detection of Ki67-positive CTCs with worse OS.	A reduction in total and PD-L1low CTCs after cycle 1 was associated with longer PFS.
Moran et al., 2022 [59] PMID: 36516370	41	IF staining (CellSieve Micro- filtration Assay)	TACs (CTCs and CAMLs), PD-L1	III	19	nivolumab, pembrolizumab, or atezolizumab	High TAC PD-L1 expression was not significantly associated with survival benefit in either ICI-treated or untreated groups.	Increase in TAC PD-L1 expression between pre-treatment and post-treatment samples was associated with better PFS.
Zhang et al., 2022 [60] PMID: 36064386	30	IF staining (IsoFlux system with CELLection Epithelial Enrich Dynabeads)	CTCs, PD-L1	V	14	sintilimab plus docetaxel	Patients with high PD-L1 expression in CTCs (CTC-PD-L1 ≥ 32.5%) had significantly better outcomes (PFS and OS).	
Park et al., 2021 [61] PMID: 34164263	83	IF staining (CD-PRIME system)	CTCs	V	15	pembrolizumab, atezolizumab		Decrease in CTC counts was associated with improved PFS and OS.
Ikeda et al., 2021 [62] PMID: 34064720	44	IF staining (Microcavity array system	CTCs, PD-L1	V	13	Nivolumab		PFS was longer in patients presenting PD-L1 positivity on CTCs ≥ 7.7% at week 8.
Dall’Olio et al., 2021 [63] PMID: 33849808	39	IF staining (CellSearch)	CTCs, PD-L1	V	15	nivolumab, pembrolizumab atezolizumab	Patients with PD-L1-positive CTCs experienced improved PFS and OS.	
Feng et al., 2021 [64] PMID: 34302805	32	CD326 (EpCAM) MicroBeads, FACSCanto™ II flow-cytometer	CTCs	V	11	cryoablation and nivolumab		CTC levels decreased in the cryoablation-nivolumab group after treatment, while no significant changes were seen in the cryoablation-only group.
Mondelo-Macia et al., 2021 [65] PMID: 34465006	50	IF staining (Parsortix system and CellSearch)	CTCs	V	18	pembrolizumab	Patients with detectable CTCs using CellSearch had significantly shorter PFS and OS than those without.	
Papadaki et al., 2020 [66] PMID: 32545559	15	IF staining (Ficoll, ISET, Parsortix)	CTCs, PD-L1, IDO	V	16	anti-PD-1 treatment	Detection of CTCs was correlated with reduced PFS while IDO-positive CTCs were associated with shorter PFS and OS.	
Alama et al., 2019 [67] PMID: 31295929	89	IHC (ScreenCell)	CTCs	V	15	nivolumab	Baseline CTC number >2 was related to worse OS.	
Tamminga et al., 2019 [68] PMID: 31291995	104	IF staining (CellSearch)	CTCs	V	18	nivolumab, ipilimumab, pembrolizumab atezolizumab	CTC presence was associated with worse OS	An increase in CTCs after 4 weeks of treatment was related to reduced PFS and OS.
Zhang et al., 2020 [69] PMID: 31678168	16	iFISH (Cytelligen)	CTCs, CTECs, PD-L1	V	16	nivolumab		Both the detection and the increase in PD-L1-positive CTECs during treatment were associated with worse PFS in patients with progressive disease.
Janning et al., 2019 [70] PMID: 31212989	11	IF staining, (Parsortix system and CellSearch)	CTCs, PD-L1	V	12	pembrolizumab, atezolizumab, nivolumab		Increase in PD-L1-positive CTCs during treatment was related to disease progression.
Dhar et al., 2018 [71] PMID: 29416054	22	IF staining (Vortex)	CTCs, PD-L1	V	13	pembrolizumab, nivolumab, avelumab, ipilimimab	PD-L1 expression > 50% PD-L1-positive CTCs was associated with improved overall response.	
Guibert et al., 2018 [72] PMID: 29748004	96	IF staining (ISET)	CTCs	V	15	nivolumab	Baseline median CTC number > 30/7.5 mL was associated with worse PFS and OS.	
Nicolazzo et al., 2016 [73] PMID: 27553175	24	IF staining (CellSearch)	CTCs, PD-L1	V	10	nivolumab		PD-L1 expression on CTCs of patients after 6 months of treatment with nivolumab could be a predictive marker.

## Data Availability

No new data were created or analyzed in this study. Data sharing is not applicable to this article.

## Supplementary Materials

The following supporting information can be downloaded at: https://www.mdpi.com/article/10.3390/ijms27125518/s1.

## Abbreviations

AF	Allele frequency
aNSCLC	Advanced non-small cell lung cancer
AUC	Area under the curve
bTMB	Blood tumour mutational burden
CAMLs	Cancer-associated macrophage-like cells
cfDNA	Cell-free DNA
chemoIO	Chemo-immunotherapy
CI	Confidence Interval
CR	Complete response
CTC	Circulating tumour cell
ctDNA	Circulating tumour DNA
CTECs	Circulating tumour endothelial cells
CTLA-4	Cytotoxic T-lymphocyte–associated protein 4
DCB	Durable clinical benefit
ddPCR	Digital droplet polymerase chain reaction
EMT	Epithelial-mesenchymal transition
ESMO	European Society for Medical Oncology
GRADE	Grading of Recommendations, Assessment, Development and Evaluations
hGE/mL	Haploid genome equivalents per millilitre
HR	Hazard ratio
ICB	Immune checkpoint blockade
ICI	Immune checkpoint inhibitor
LB	Liquid biopsy
LC	Lung cancer
LOD	Limit of detection
LoE	Level of evidence
MAF	Mutant allele frequency/fraction
maxMAF	Maximal mutant allele frequency/fraction
MMPM	Mutant molecules per millilitre
MRD	Minimal residual disease
MSAF	Maximum somatic allele frequency
MTM	Mean tumour molecules
NGS	Next generation sequencing
NSCLC	Non-small cell lung cancer
OCEBM	Oxford Centre for Evidence-Based Medicine
OS	Overall survival
PD-1	Programmed cell death protein 1
PD-L1	Programmed death ligand 1
PFS	Progression-free survival
PR	Partial response
PRISMA	Preferred reporting items for systematic reviews and meta-analyses
QUIPS	Quality In Prognosis Studies
RECIST	Response Evaluation Criteria in Solid Tumours
REMARK	Reporting Recommendations for Tumor Marker Prognostic Studies
REML	Restricted maximum likelihood
RR	Response rate
SCNA	Somatic copy number alteration
sWGS	Shallow whole-genome sequencing
TACs	Tumour-associated cells
TFx	Tumour fraction
TKI	Tyrosine kinase inhibitor
TMB	Tumour mutational burden
VAF	Variant allele frequency
